# Using New and Emerging Technologies to Identify and Respond to Suicidality Among Help-Seeking Young People: A Cross-Sectional Study

**DOI:** 10.2196/jmir.7897

**Published:** 2017-07-12

**Authors:** Frank Iorfino, Tracey A Davenport, Laura Ospina-Pinillos, Daniel F Hermens, Shane Cross, Jane Burns, Ian B Hickie

**Affiliations:** ^1^ Brain and Mind Centre The University of Sydney Sydney Australia

**Keywords:** suicidal ideation, mental health, primary health care, telemedicine, health services

## Abstract

**Background:**

Suicidal thoughts are common among young people presenting to face-to-face and online mental health services. The early detection and rapid response to these suicidal thoughts and other suicidal behaviors is a priority for suicide prevention and early intervention efforts internationally. Establishing how best to use new and emerging technologies to facilitate person-centered systematic assessment and early intervention for suicidality is crucial to these efforts.

**Objective:**

The aim of this study was to examine the use of a suicidality escalation protocol to respond to suicidality among help-seeking young people.

**Methods:**

A total of 232 young people in the age range of 16-25 years were recruited from either a primary mental health care service or online in the community. Each young person used the Synergy Online System and completed an initial clinical assessment online before their face-to-face or online clinical appointment. A suicidality escalation protocol was used to identify and respond to current and previous suicidal thoughts and behaviors.

**Results:**

A total of 153 young people (66%, 153/232) reported some degree of suicidality and were provided with a real-time alert online. Further levels of escalation (email or phone contact and clinical review) were initiated for the 35 young people (15%, 35/232) reporting high suicidality. Higher levels of psychological distress (*P*<.001) and a current alcohol or substance use problem (*P*=.02) predicted any level of suicidality compared with no suicidality. Furthermore, predictors of high suicidality compared with low suicidality were higher levels of psychological distress (*P*=.01), psychosis-like symptoms in the last 12 months (*P*=.01), a previous mental health problem (*P*=.01), and a history of suicide planning or attempts (*P*=.001).

**Conclusions:**

This study demonstrates the use of new and emerging technologies to facilitate the systematic assessment and detection of help-seeking young people presenting with suicidality. This protocol empowered the young person by suggesting pathways to care that were based on their current needs. The protocol also enabled an appropriate and timely response from service providers for young people reporting high suicidality that was associated with additional comorbid issues, including psychosis-like symptoms, and a history of suicide plans and attempts.

## Introduction

Suicidal thoughts are common among young people presenting to traditional face-to-face mental health services and engaging with online mental health services [1,2]. Young people presenting to such services are also more likely to engage in suicidal behaviors (such as planning or attempts), which are among the strongest predictors of completed suicide [3-6]. Suicidal thoughts and behaviors are also associated with complex comorbid mental health problems [7], alcohol, or other substance use problems [8], as well as social and economic difficulties that contribute to greater disability [9]. Together, this highlights the need for suicide prevention and early intervention strategies that facilitate the early detection and rapid response to suicidality for those help-seeking young people [10,11].

This is a particularly pertinent issue given that almost half of those who have died by suicide had contact with a primary care provider within one month before committing suicide [12], and one-quarter of those with depression who die by suicide are likely to have been in active engagement with mental health services at the time of death [13-16]. This emphasizes the challenge mental health professionals and services face when trying to identify and respond to those at high risk of engaging in harmful suicidal behaviors. This may be influenced by the relatively limited exposure to acutely suicidal patients in a clinician’s daily work and a lack of systematic or organizational processes that directly address suicidal thoughts and behaviors [17].

New and emerging technologies (eg, mobile and Internet-based apps and e-tools) may be able to improve the systematic assessment and response to suicidality at a service and individual level so that those at risk can receive the appropriate care sooner [18,19]. Evidence indicates that online assessments are preferred and accurate for identifying suicidal thoughts and behaviors and other sensitive information [20,21], and online screening has demonstrated utility for facilitating access to treatment, especially when integrated with professional services [22-24]. The integration of these technologies with traditional services is crucial, and understanding how best to utilize the benefits of new and emerging technologies in terms of accessibility is an important goal for the ongoing development of effective early intervention strategies that target suicidal thoughts and behaviors.

The aim of this study was to examine the use of a suicidality escalation protocol embedded within the Synergy Online System (Textbox 1) that identifies and responds to suicidal thoughts and behaviors experienced by young people (aged 16-25 years) seeking help through primary mental health care and community settings and to identify specific predictors of suicidality.

**Synergy Online System.**

The Synergy Online System is a personalized Internet-based resource designed to help people manage their physical, mental, and social wellbeing using a mixture of evidence-based apps, e-tools, and online and face-to-face services. One of the cornerstone principles of the Synergy Online System is a focus on the entire spectrum of health and well-being, from those who simply want to achieve goals to improve their daily habits, to those experiencing serious mental health problems. A key feature of the Synergy Online System is that it’s configurable (ie, can rearrange or turn on or off different components within the system as well as tailor content), which allows it to easily adapt and thus meet the needs of end users. The System aims to transform the provision of mental health services by delivering readily accessible, affordable, and equitable mental health care through an increased focus on prevention and early intervention and improving the management of mental disorders across settings.

## Methods

### Participants

Participants in this study included young people aged 16-25 years who had access to the Internet and were either seeking help through primary mental health care services (*headspace*) or online in the community for the first time. Participants were recruited into one of three groups as follows:

Primary care sample 1: Participants were recruited from a group of young people presenting for the first time to *headspace* Camperdown or *headspace* Campbelltown (both in Sydney, Australia) from July 2015 to July 2016. These participants were recruited for the initial “proof of concept” trial of the Mental health eClinic (MHeC) of the Synergy Online System.

Primary care sample 2: Participants were recruited from a group of young people presenting for the first time to any *headspace* service in the Central and Eastern Sydney Primary Health Network (ie, Ashfield, Bondi Junction, Camperdown, Hurstville, and Miranda) from September 2016 to February 2017. These participants were recruited for a trial of the MHeC of the Synergy Online System embedded with primary mental health care services (*headspace*).

Community sample: Participants were recruited from three urban, regional, and rural communities in New South Wales that have a number of geographical, social, and economic vulnerabilities (ie, Central Coast, Western Sydney, and the Far West). Participants were recruited through targeted advertising in each of these communities (including posters and postcards in local businesses, paid Facebook advertisements, and advertisements on organizational social media channels) from March 2016 to June 2016. Young people were invited to participate in the study if they were currently living in one of these communities and had regular access to a mobile phone and the Internet.

### Ethics

The University of Sydney Human Research Ethics Committees approved these studies and all participants gave written or online informed consent when they first accessed the Synergy Online System and before completing the initial clinical assessment.

### Measures

All participants were invited to complete an initial clinical assessment (accessed via the MHeC of the Synergy Online System). Participants from primary care sample 1 were provided with a URL to the alpha version of the MHeC and asked to complete the initial clinical assessment online before either a video visit or face-to-face appointment with a clinician. Participants from primary care sample 2 were provided with a URL to the beta version of the MHeC (with the video visit “turned off”) and asked to complete the initial assessment before their scheduled face-to-face appointment with a clinician. Participants from the community sample either navigated themselves to the MHeC or were automatically directed (via an e-tool embedded within the Synergy Online System) to the beta version of the MHeC (with the video visit “turned on”) if they were expressing psychological distress. For all participants using the MHeC, a “need help now” button was always displayed to provide the details of relevant emergency and helpline services for those who sought immediate help.

The initial clinical assessment assesses a range of mental health outcomes, as well as comorbid and associated risk factors. Being administered online and using smart skips, the full assessment takes approximately 45 min to complete (median, 47.5 min) and includes 14 modules (in the following order): demographics; current education and employment participation; mental health concerns; self-harm and suicidal behaviors; tobacco, alcohol, and other substance use; physical activity; sleep-wake behaviors; lifetime disorders; physical and mental health history; cognition; eating behaviors and body image; social connectedness; and puberty. Participants completed all modules. For the purposes of this study, the following measures were specifically selected and included for analysis.

### Demographics

Participants’ age, gender, highest level of education, and current education, employment, and training status (used to determine not in education, employment or training [NEET] status).

### Mental Health

Current psychological distress was assessed using the Kessler-10 (K10) questionnaire [25] that is a well-validated measure of general psychological symptoms and distress widely used in adult and adolescent populations in both clinical and community settings. Hypomania-like symptoms over the last 12 months were assessed using a screener derived from the Altman self-rating scale [26]. Psychosis-like symptoms over the last 12 months were assessed using a screener derived from Community Assessment of Psychotic Experiences-Positive Symptoms scale [27]. Participants were also asked “Have you ever experienced a major mental health or behavioral problem that has affected your everyday life?” and this was used as a proxy for a previous mental health problem.

#### Suicidality

The Suicide Ideation Attributes Scale (SIDAS) is a 5-item scale assessing suicidal ideation over the past month [28]. The scale assesses frequency, controllability, closeness to attempt, distress, and interference with daily activities on a 10-point Likert scale. A score of 0 corresponds to “no current ideation”, a score of 1 to 20 corresponds to “low current suicidal ideation”, and a score of 21 to 50 corresponds to “high current suicidal ideation”. The scale has strong internal reliability (Cronbach alpha=.91). Lifetime suicidal thoughts and behaviors (ideation, planning, and attempts) were assessed by three questions from the Youth Risk Behaviors Survey [29,30]; (1) “Have you ever seriously thought about killing yourself?” (2) (1) “Have you ever seriously thought about killing yourself?” (2) “Have you ever made a plan about how you would kill yourself?” and (3) “How many times have you actually tried to kill yourself?”.

#### Functioning

An item from the Brief Disability Questionnaire (BDQ) was used to assess participant’s inability to carry out daily tasks over the previous month [31]. Specifically, participants were asked “Over the past month, how many days in total were you unable to carry out your usual daily activities fully?” This enabled a calculation of “days out of role in the past month.”

#### Alcohol and Substance Use

Two questions about alcohol and substance use were used to assess the presence of a current comorbid alcohol or substance use problem. Specifically, participants were asked “Have you recently thought that you should cut down on alcohol or other addictive drugs?” (derived from the CAGE questionnaire; [32]) and “Have you recently had a friend, relative or doctor suggest that you should cut down on alcohol or other addictive drugs?” (derived from the Alcohol Use Disorders Identification Test; [33]). Participants who answered “no” to one or both of these questions were categorized as “no problem”, and participants who answered “yes” to both questions were categorized as “likely problem” [34].

### Statistical Analyses

All statistical analyses were performed using Statistical Package for the Social Sciences (SPSS 22.0 for Windows). Group differences between the three sample groups (primary care sample 1, primary care sample 2, and community sample) were assessed using the Kruskal-Wallis H test for continuous variables and the chi-square test for categorical variables. The sample was then split by suicidality group (see Textbox 2; “no suicidality”, “low suicidality”, and “high suicidality”) to assess group differences using the Kruskal-Wallis H test for continuous variables and the chi-square test for categorical variables. To examine the independent predictors of suicidality, two separate logistic regressions were conducted. The first model compares the “no suicidality” group with the “any suicidality” group (low and high suicidality groups combined). The second model compares the “low suicidality” and “high suicidality” groups. For both models, variables were entered using a forward forced-entry method with demographic variables (age, gender, education, and NEET status) entered in the first block, current mental health variables (K10, hypomania-like symptoms, psychosis-like symptoms, and alcohol or substance use) entered in the second block, mental health history variables (previous mental health problem, suicide plans or attempts history) entered in the third block, and functioning (days out of role) entered in the final block. To control for sample groups in the analyses, “sample” was also entered in the final block. Only models with nonsignificant Hosmer-Lemeshow goodness of fit tests were included.

**Suicidality escalation protocol.**

**Figure 1:**
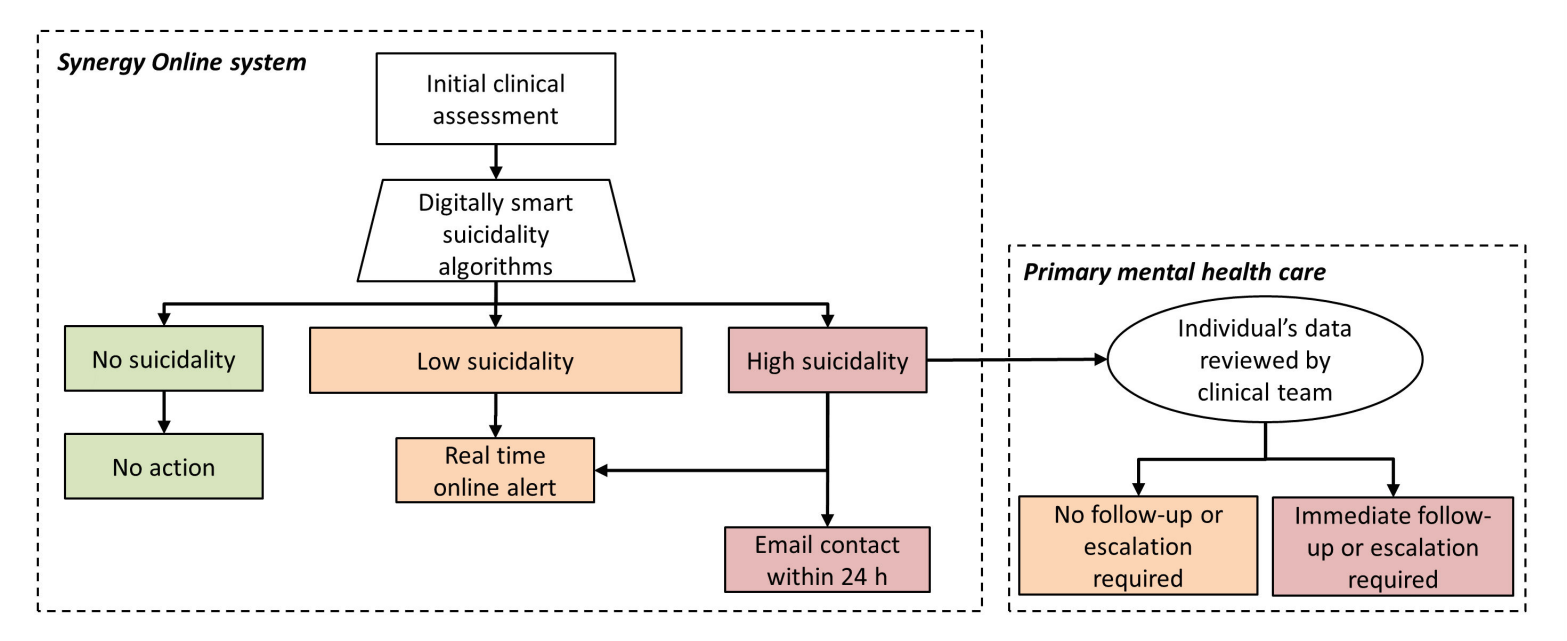
Suicidality escalation protocol.

The suicidality escalation protocol involves multiple levels of action, dependent on the participants’ responses to the initial assessment (Figure 1). Every young person completes the initial clinical assessment, and at the end of the suicidality module the digitally smart algorithms assess current and past suicidality. The algorithm assigns them to one of the three groups: “no suicidality” (SIDAS score of 0 and no lifetime suicidal behaviors), “low suicidality” (SIDAS score of 1-20 and/or lifetime suicidal behaviors), and “high suicidality” (SIDAS score of 21 to 50). For the “no suicidality” group, no action is taken. For those in the “low suicidality” or “high suicidality” groups, an automatic real-time alert is immediately presented on the young person’s screen. The alert displays information regarding both crisis and non-crisis services so the young person can access immediate support if needed. For those in the “high suicidality” group, two additional actions are initiated. First, a notification is sent to the clinical research team who initiate email contact with the participant within 24 h. This email aims to provide further information that encourages the young person to seek help and requests they inform the clinical research team how they are going by replying to the email or calling. Second, for those currently in contact with a service, the young person’s data is forwarded to the clinical service for review, and a decision is made regarding further follow-up or escalation. Further follow-up or escalation involves one or more of the following: contact over the phone, rescheduling the young person’s appointment or an online “video visit” within the subsequent 72 h. Importantly, this suicidality escalation protocol is designed to respond in real-time to the suicidality expressed by the young person and is not used to determine suicide risk. Formal suicide risk is determined by a health professional or a multidisciplinary team after making contact with the young person and reviewing the young person’s data.

## Results

### Sample Characteristics

The demographic and behavioral characteristics for each sample are presented in Tables 1 and 2. A total of 232 participants were included in the analyses (95 from primary care sample 1, 105 from primary care sample 2, and 32 from the community sample). The mean age of the entire sample was 20.44 years (standard deviation [SD]=2.59; median=21 years), 69% (160/232) were female, and 37% (87/232) were classified as NEET. Across all three samples, the mean K10 score was in the severe range of psychological distress (x̅=28.99, SD=8.86; median=30); 17% (40/232) reported no psychological distress, 13% (31/232) reported mild psychological distress, 19% (43/232) reported moderate psychological distress, and 51% (118/232) reported severe psychological distress. The mean SIDAS score was 8.25 (SD=11.52; median=2); 39% (90/232) reported no current suicidal ideation, 46% (107/232) reported low current suicidal ideation, and 15% (35/232) reported high current suicidal ideation. The only statistically significant differences identified between the three sample groups were for “days out of role” over the past month (χ^2^_2_=16.2, *P*<.001).

### Suicidality Escalation in Primary Care—A Proof of Concept

The first use of the suicidality escalation protocol in a primary mental health care setting occurred at *headspace* Camperdown and *headspace* Campbelltown and was rolled out entirely by the clinical research team. Of the entire primary care sample 1, 33% (31/95) were identified as “no suicidality” and so no action was initiated, 51% (49/95) were identified as “low suicidality” and were presented with a real-time alert only, and 16% (15/95) were identified as “high suicidality,” which initiated the real-time alert and an additional two escalation actions. All 15 individuals were contacted via email by the clinical research team and had their data reviewed. Of these 15, 7 had their entry into clinical care escalated (ie, their initial clinical assessment appointment was brought forward). Clinicians reported that the decision to escalate an individual was influenced by the following: (1) concerns over specific suicidal ideation attributes such as little of control over suicidal thoughts (5/7 participants) and closeness to making an attempt (5/7 participants), (2) concerns over the presence of hypomania or psychosis-like symptoms (1/7 participants), (3) recent plans to make an attempt that were identified upon follow-up (1/7 participants), (4) few protective factors identified upon follow-up (1/7 participants), (4) few protective factors identified at follow-up (1/7 participants), and (5) recent self-harm (1/7 participants). All 7 participants were escalated due to one or more of these factors being present, and the clinician decided that their initial appointment for care was too long to wait. The remaining 8 participants did not have the initial assessment appointment brought forward due to most, or all of the above, factors being absent or because their clinical appointment was scheduled within a few days (range=0-5 days), which was deemed to be sufficient by the reviewing clinician.

**Table 1:** Demographic characteristics by sample group (N=232).

Characteristics		Primary care 1 (n=95)	Primary care 2 (n=105)	Community (n=32)	*P* value
Age, mean (SD^a^)		20.39 (2.56)	20.41 (2.53)	20.66 (2.90)	.88
**Gender, n (%)**					.76
	Female	68 (72)	71 (68)	21 (66)	
	Male	27 (28)	34 (32)	11 (34)	
**Education^b^, n (%)**					.70
	Secondary	44 (46)	49 (48)	16 (55)	
	Tertiary	51 (54)	54 (52)	13 (45)	
**NEET^c^ status, n (%)**					.12
	Non-NEET	62 (65)	59 (56)	24 (75)	
	NEET	33 (35)	46 (44)	8 (25)	

^a^SD: standard deviation.

^b^“no formal education” and “primary education” groups were left out due to insufficient cell counts (n=5 cases missing).

^c^NEET: not in education, employment or training.

### Suicidality Escalation Scaled Up for Use in Primary Care

The suicidality escalation protocol was scaled up and rolled out across all *headspace* services in the Central and Eastern Sydney Primary Health Network. Of the entire primary care sample 2, 34% (36/105) were identified as “no suicidality” and so no action was initiated, 55% (57/105) were identified as “low suicidality” and were presented with a real-time alert only, and 11% (12/105) were identified as “high suicidality,” which initiated the real-time alert and an additional two escalation actions. All 12 individuals were contacted via email by the clinical research team and had their data forwarded for review to the clinical service responsible so that specific service protocols could be initiated.

Of the entire community sample, 37.5% (12/32) young people were identified as “no suicidality” and so no action was initiated, 37.5% (12/32) were identified as “low suicidality” and were presented with a real-time alert, and 25% (8/32) were identified as “high suicidality”, which initiated the real-time alert and an additional two escalation actions. All 8 individuals were contacted via email by the clinical research team, and had their data reviewed.

**Table 2:** Behavioral characteristics by sample group (N=232).

Characteristics		Primary care 1 (n=95)	Primary care 2 (n=105)	Community (n=32)	*P* value
K10^a^ score, mean (SD^b^)		29.28 (8.16)	29.75 (8.28)	25.59 (11.76)	.11
**K10 category, n (%)**					.08
	No	13 (14)	15 (14)	12 (38)	
	Mild	15 (16)	13 (13)	3 (9)	
	Moderate	18 (19)	20 (19)	5 (16)	
	Severe	49 (51)	57 (54)	12 (37)	
SIDAS^c^ score, mean (SD)		7.93 (11.50)	7.52 (9.71)	11.59 (16.05)	.87
**SIDAS category, n (%)**					.34
	No ideation	37 (39)	40 (38)	13 (41)	
	Low ideation	43 (45)	53 (51)	11 (34)	
	High ideation	15 (16)	12 (11)	8 (25)	
**Hypomania-like symptoms, last 12 months, n (%)**					.06
	No	68 (72)	88 (84)	22 (69)	
	Yes	27 (28)	17 (16)	10 (31)	
**Psychosis-like symptoms, last 12 months, n (%)**					.51
	No	65 (68)	71 (68)	25 (78)	
	Yes	30 (32)	34 (32)	7 (22)	
Days out of role in past month, mean (SD)		7.53 (7.22)	8.04 (8.47)	2.34 (2.66)	<.001
**Alcohol and substance use, current, n (%)**					.22
	No problem	60 (73)	87 (83)	25 (78)	
	Likely problem	26 (27)	18 (17)	7 (22)	
**Previous mental health problem, ever, n (%)**					.24
	No	27 (28)	31 (29)	14 (44)	
	Yes	68 (72)	74 (71)	18 (56)	
**Suicide plans or attempts, ever, n (%)**					.18
	No	67 (71)	66 (63)	17 (53)	
	Yes	28 (29)	39 (37)	15 (47)	

^a^K10: Kessler-10.

^b^SD: standard deviation.

^c^SIDAS: Suicide Ideation Attributes Scale.

### Predictors of Suicidality

The overall sample was split according to “no suicidality”, “low suicidality”, and “high suicidality” to examine the demographic and behavioral differences between these groups (Tables 3 and 4). No differences were identified between the sample groups (*P*=.33) or for the demographic variables; age (*P*=.08), gender (*P*=.74), or NEET status (*P*=.29); however, significant differences were identified for highest level of education (χ^2^_2_=8.6, *P*=.01). In terms of behavioral characteristics, no differences were identified for days out of role (*P*=.09); however, significant differences were identified between the three suicidality groups for psychological distress (χ^2^_2_=48.5, *P*<.001), hypomania-like symptoms in the last 12 months (χ^2^_2_=12.9, *P*=.002), psychosis-like symptoms in the last 12 months (χ^2^_2_=29.2, *P*<.001), alcohol or substance use (χ^2^_2_=8.3, *P*=.02), and previous mental health problem (χ^2^_2_=15.8, *P*<.001). Significant differences between the “low suicidality” and “high suicidality” groups were also identified for history of suicide plans or attempts (χ^2^_1_=22.3, *P*<.001).

**Table 3:** Demographic characteristics by suicidality group (N=232).

Characteristics		Suicidality			*P* value
		No (n=79)	Low (n=118)	High (n=35)	
**Sample, n (%)**					.33
	Primary care 1	31 (39)	49 (42)	15 (43)	
	Primary care 2	36 (46)	57 (48)	12 (34)	
	Community	12 (15)	12 (10)	8 (23)	
Age, mean (SD)^a^		20.32 (2.66)	20.75 (2.52)	19.66 (2.53)	.08
**Gender, n (%)**					.74
	Female	57 (72)	79 (67)	24 (69)	
	Male	22 (28)	39 (33)	11 (31)	
**Education^b^, n (%)**					.01
	Secondary	32 (41)	53 (46)	24 (71)	
	Tertiary	46 (59)	62 (54)	10 (29)	
**NEET^c^ status, n (%)**					.29
	Non-NEET	48 (61)	71 (60)	26 (74)	
	NEET	31 (39)	47 (40)	9 (26)	

^a^SD: standard deviation.

^b^“no formal education” and “primary education” groups were left out due to insufficient cell counts (n=5 cases missing).

^C^NEET: not in education, employment or training.

**Table 4:** Behavioral characteristics by suicidality group (N=232).

Characteristics		Suicidality			*P* value
		No (n=79)	Low (n=118)	High (n=35)	
**Sample, n (%)**					.33
	Primary care 1	31 (39)	49 (42)	15 (43)	
	Primary care 2	36 (46)	57 (48)	12 (34)	
	Community	12 (15)	12 (10)	8 (23)	
K10^a^ score, mean (SD^b^)		24.30 (8.04)	29.92 (8.22)	36.43 (6.41)	<.001
**K10 category, n (%)**					<.001
	No	26 (33)	14 (12)	0 (0)	
	Mild	14 (18)	16 (14)	1 (3)	
	Moderate	17 (21)	22 (18)	4 (11)	
	Severe	22 (28)	66 (56)	30 (86)	
**Hypomania-like symptoms, last 12 months, n (%)**					.002
	No	67 (85)	92 (78)	19 (54)	
	Yes	12 (15)	26 (22)	16 (46)	
**Psychosis-like symptoms, last 12 months, n (%)**					<.001
	No	67 (85)	82 (70)	12 (34)	
	Yes	12 (15)	36 (30)	23 (66)	
Days out of role in past month, mean (SD)		6.22 (7.63)	7.18 (7.69)	8.46 (7.35)	.09
**Alcohol and substance use, current, n (%)**					.02
	No problem	70 (89)	87 (74)	24 (69)	
	Likely problem	9 (11)	31 (26)	11 (31)	
**Previous mental health problem, ever, n (%)**					<.001
	No	34 (43)	36 (30)	2 (6)	
	Yes	45 (57)	82 (70)	33 (94)	
**Suicide plans or attempts, ever, n (%)**					<.001^c^
	No	79 (100)	67 (57)	4 (11)	
	Yes	0 (0)	51 (43)	31 (89)	

^a^K10: Kessler-10.

^b^SD: standard deviation.

^c^This *P* value refers to the 2x2 comparison between the low and high groups. By definition the “no suicidality” group has 0 “yes” responses.

Further analyses using logistic regression were conducted to (1) identify predictors of “no suicidality” compared with “any suicidality” (low and high suicidality groups combined) (Model 1, Table 5), and (2) to identify predictors of “low suicidality” compared with “high suicidality” (Model 2, Table 5). Model 1 identified that higher psychological distress and a current alcohol or substance use problem were predictors of “any suicidality” compared with “no suicidality” (χ^2^_12_=57.7, *P*<.001, R^2^=0.22). Model 2 identified that higher psychological distress, any psychosis-like symptoms in the last 12 months, a previous mental health problem, and a history of suicide plans or attempts were all predictors of “high suicidality” compared with “low suicidality” (χ^2^_13_=67.0, *P*<.001, R^2^=0.36).

**Table 5:** Logistic regression models showing predictors of suicidality (N=232).

		No suicidality versus any suicidality^a^			Low suicidality versus high suicidality^b^		
		Beta (SE^c^)	OR (95% CI)	*P* value	Beta (SE)	OR (95% CI)	*P* value
Age		.05 (0.07)	1.05 (0.91-1.22)	.47	−.17 (0.15)	0.84 (0.63-1.13)	.26
**Gender**							
	Female		1.00			1.00		
	Male	.65 (0.38)	1.92 (0.92-4.02)	.08	.66 (0.64)	1.94 (0.55-6.81)	.30
**Education^d^**							
	Secondary		1.00			1.00	
	Tertiary	−.22 (0.39)	0.81 (0.37-1.75)	.58	−.91 (0.69)	0.40 (0.10-1.57)	.19
**NEET^e^ status**							
	NEET		1.00			1.00	
	Non-NEET	.30 (0.35)	1.35 (0.68-2.67)	.39	.92 (0.68)	2.50 (0.66-9.51)	.18
K10^f^ score		.11 (0.02)	1.12 (1.07-1.17)	<.001	.11 (0.04)	1.12 (1.03-1.21)	.01
**Hypomania-like symptoms, last 12 months**							
	No		1.00			1.00	
	Yes	.14 (0.45)	1.16 (0.48-2.76)	.75	.41 (0.60)	1.50 (0.47-4.84)	.50
**Psychosis-like symptoms, last 12 months**							
	No		1.00			1.00	
	Yes	.80 (0.41)	2.22 (1.00-4.95)	.05	1.54 (0.58)	4.68 (1.51-14.53)	.01
**Alcohol and substance use, current**							
	No problem		1.00			1.00	
	Likely problem	1.04 (0.46)	2.84 (1.15-7.05)	.02	−.12 (0.63)	0.89 (0.26-3.04)	.85
**Previous mental health problem**							
	No		1.00			1.00	
	Yes	.42 (0.35)	1.52 (0.77-3.03)	.23	2.43 (0.99)	11.34 (1.64-78.30)	.01
**Suicide plans or attempts, ever**							
	No					1.00	
	Yes	N/A^g^	N/A	N/A	2.34 (0.70)	10.41 (2.65-40.83)	.001
Days out of role, past month		−.02 (0.02)	0.98 (0.94-1.03)	.42	.01 (0.04)	1.00 (0.92-1.09)	.93
**Sample**							
	Community		1.00			1.00	
	Primary care 1	−.22 (.57)	0.80 (0.26-2.43)	.69	.25 (0.86)	1.28 (0.24-6.83)	.77
	Primary care 2	−.27 (.57)	0.76 (0.25-2.30)	.63	−.51 (0.92)	0.60 (0.10-3.67)	.58

^a^Model 1 **:** R^2^=0.22 (Cox and Snell), 0.31 (Nagelkerke). Model χ^2^_12_=57.7, *P*<.001.

^b^Model 2 **:** R^2^=0.36 (Cox and Snell), 0.55 (Nagelkerke). Model χ^2^_13_=67.0, *P*<.001.

^c^SE: standard error.

^d^“no formal education” and “primary education” groups were left out due to insufficient cell counts (n=5 cases missing) *.*

^e^NEET: not in education, employment or training.

^f^K10: Kessler-10.

^g^N/A: Not applicable, this comparison is invalid since the “no suicidality” group, by definition, has no history of suicide plans or attempts and therefore was left out of the model.

## Discussion

### Principal Findings

We identified that two-thirds of help-seeking young people reported some degree of suicidality, and the protocol provided these young people with a real-time alert online. Further levels of escalation (email or phone contact and clinical review) were initiated for the 15% (35/232) of young people who reported high suicidality. Higher levels of psychological distress and a current alcohol or substance use problem predicted any level of suicidality (compared with no suicidality). In addition to higher levels of psychological distress, psychosis-like symptoms in the last 12 months, a previous mental health problem, and a history of suicide plans or attempts were specific predictors of high suicidality (compared with low suicidality). These results support the use of new and emerging technologies to facilitate the systematic assessment and detection of young people experiencing suicidal thoughts with additional comorbidities and enable an appropriate and timely response from service providers.

The use of the suicidality escalation protocol of the Synergy Online System as an adjunct to traditional primary mental health care services assisted clinical decision-making about suicide risk and the need for care among those young people reporting higher levels of suicidality. Of the young people in primary care, 13.5% (27/200) had their case escalated to clinical review by a clinician or clinical team before their entry into care. Importantly, none of these young people were referred to crisis services but instead had their entry into care facilitated due to a clinically perceived higher need for immediate care. This escalation process ensured that individuals presenting to primary care services with increased suicidality were not delayed by a service waitlist, which commonly arises from a mismatch between service demand and capacity [35]. Instead, the Synergy Online System was able to deploy many immediate actions to ensure the suicidality risk is addressed in a timely and efficient manner. The use of this System has already had major implications on actual health service practices for the youth mental health services that have adopted Synergy; specifically, improving patient and workforce management through systematic assessment, automatic escalation of an individual’s data, and assisting clinical team review and decision-making processes.

Importantly, the results here also highlight the benefits of offering online services to young people by allowing mental health care and the service to be brought to the young person when they need it, wherever they live, rather than relying on young people to present initially to a face-to-face service which has many barriers to overcome [36]. Notably, there were comparable levels of suicidal ideation in the community sample compared with those presenting to primary mental health care. These young people may never have presented to a face-to-face service either because of common barriers to help-seeking or because a service was not available locally [37,38]. The use of the online service meant that a service could “come to them” when they needed it and in a manner that is preferable to some young people [39]. The use of new and emerging technologies as reported in this paper is critical in reaching the high numbers of at-risk youth in the community who are not presenting to traditional face-to-face services. Importantly, with the rapid increase in new and emerging technologies for mental health care, there is a significant need for effective suicide escalation protocols that can appropriately and efficiently manage risk. A real-time mapping system to (local) mental health services might be useful for those in the community who seek help online to ensure the system effectively facilitates help-seeking behavior, which is a crucial unresolved issue for online assessment and feedback systems [40-42]. Similarly, further follow-up through partnerships with specific local or national suicide prevention organizations may be needed to increase help-seeking behavior for those identified as at-risk or in need of care in the community.

Psychological distress differentiated between each level of suicidality identified, which is consistent with the established relationship between distress and suicidality [43,44]. The only other predictor that differentiated between no suicidality and any suicidality was a current alcohol or substance use problem. This reflects the common relationship between alcohol and substance use and suicidal thoughts and behaviors, particularly among young people with mental health problems [45]. Young people reporting high suicidality were also more likely to report psychosis-like symptoms in the last 12 months, a previous mental health problem, and a history of suicide plans or attempts. Together, this confirms the significant comorbidity that help-seeking young people initially present with and reiterates the need for services to be equipped to respond to the differing individual needs a young person has when they first present to care.

The ongoing development of the Synergy Online System would benefit from employing methodologies that utilize longitudinal outcomes to improve the existing algorithms accuracy for identifying individual cases of suicidality that should be escalated and followed up immediately by a clinician and service. Machine learning methodologies are increasingly used in psychiatric research as they facilitate individual-level prediction of unseen observations, which makes them suitable for the development of clinically useful digital tools [46]. Recent evidence has demonstrated the use of these algorithms to utilize clinical and demographic variables to predict suicide attempters among a group of mood disorder patients with accuracy comparable with most breast cancer prediction algorithms [47,48], whereas another study demonstrated the utility of such algorithms to differentiate between suicidal and nonsuicidal patients [49]. Employing these approaches could improve the personalization of care beyond simple cut-off scores and include key risk factors specific to a particular individual. Similar approaches have been employed by Facebook who have developed an online tool that uses machine learning to identify users at risk of suicide by assessing their posts and comments and provides the user with a number of options for how to get help [50]. These semiautomated approaches require rigorous evaluation and validation using qualitative person-centered approaches such as user acceptance testing, in addition to more traditional quantitative methods to determine whether they are appropriate and effective. This is important for the development of clinically useful and scalable suicide prevention and early intervention efforts that are integrated with existing services and practices.

### Limitations

For the future development of the protocol, some limitations need to be addressed. First, the initiation of the suicidality escalation protocol is dependent on when the young person completes the online assessment. So young people at-risk who don’t complete the online assessment immediately cannot be identified and spend a longer period under distress and not in care. Second, the outcome for those who had their entry into care escalated is unclear, so it is difficult to determine the impact of the suicidality escalation protocol on their clinical outcome. This was beyond the scope of this particular study, but it is an important focus for future research to establish the long term impact of this protocol on engagement with services and clinical trajectory. Another key focus for this work would be to determine whether the protocol missed individuals who would become high risk or later engage in suicidal behaviors. Third, the relatively small sample size of the community sample, compared with the two primary care sample groups, means that the sample characteristics were somewhat biased toward the primary care groups and limits the generalizability of these results to young people in the community who seek help online. Finally, the use of the K10 as a measure of general psychological distress may be limited primarily to depression and anxiety symptoms and less useful for other mental health problems common in adolescence.

### Conclusions

This study contributes to the research and knowledge about the use of new and emerging technologies to identify and respond to increased suicidality among help-seeking young people. Young people with increased suicidality were more likely to present with a number of comorbid issues including psychosis-like symptoms and a history of plans or attempts, which emphasizes the need for these young people to receive appropriate and timely care.

## Abbreviations

BDQ: Brief Disability Questionnaire
K10: Kessler-10 Questionnaire
MHeC: Mental health eClinic
NEET: Not in Educational, Employment or Training
SIDAS: Suicide Ideation Attributes Scale
SPSS: Statistical Package for the Social Sciences
