# Correction of: Using New and Emerging Technologies to Identify and Respond to Suicidality Among Help-Seeking Young People: A Cross-Sectional Study

**DOI:** 10.2196/jmir.8804

**Published:** 2017-10-30

**Authors:** Frank Iorfino, Tracey A Davenport, Laura Ospina-Pinillos, Daniel F Hermens, Shane Cross, Jane Burns, Ian B Hickie

**Affiliations:** ^1^ Brain and Mind Centre The University of Sydney Sydney Australia

In the paper by Frank Iorfino et al, “Using New and Emerging Technologies to Identify and Respond to Suicidality Among Help-Seeking Young People: A Cross-Sectional Study” (J Med Internet Res 2017;19(7):e247), a mistake was made in the final stage of copy editing. In the Introduction, the first sentence of the second paragraph should have appeared as follows: “This is a particularly pertinent issue given that almost half of those who have died by suicide had contact with a primary care provider within one month of the suicide [12], and one-quarter of those with depression who die by suicide are likely to have been in active engagement with mental health services at the time of death [13-16].”

Instead of the above, the first part of the sentence was incorrectly worded as: “This is a particularly pertinent issue given that almost half of those who have died by suicide had contact with a primary care provider within one month before *committing suicide* [12]...” (emphasis added). The phrase “committing suicide” was chosen by the proofreader without realizing that it can be perceived as stigmatizing and at times offensive in the mental health field. We regret this error and have updated our copyediting guidelines to reflect the recommended terminology [1].

Another minor error was introduced in the section, “Suicidality Escalation in Primary Care—A Proof of Concept.” In the first paragraph, where factors reported by clinicians as influencing the decision to escalate an individual are given, the “(4)” was repeated. The list should have appeared as follows: (1) concerns over specific suicidal ideation attributes such as little of control over suicidal thoughts (5/7 participants) and closeness to making an attempt (5/7 participants), (2) concerns over the presence of hypomania or psychosis-like symptoms (1/7 participants), (3) recent plans to make an attempt that were identified upon follow-up (1/7 participants), (4) few protective factors identified upon follow-up (1/7 participants), (5) few protective factors identified at follow-up (1/7 participants), and (6) recent self-harm (1/7 participants). 

The corrected article will appear in the online version of the paper on the JMIR website on October 30, 2017, together with the publication of this correction notice. Because this was made after submission to PubMed Central, the corrected article will also be re-submitted to PubMed Central.
